# Intramedullary neurotuberculoma in a patient with disseminated tuberculosis: A case report and a narrative review

**DOI:** 10.1016/j.radcr.2026.07.076

**Published:** 2026-08-18

**Authors:** Diego Belandrino Swerts, Carolina Marano, Narriman Hazime, Gabrielle Silva, Taiana Cunha Ribeiro, Augusto Cesar Penalva de Oliveria

**Affiliations:** Neurology Department, Irmandade Santa Casa de Misericórdia de São Paulo, São Paulo, Brazil

**Keywords:** Neurology, Intramedullary tuberculoma, Tuberculosis, Transverse myelitis

## Abstract

Tuberculosis has a wide spectrum of clinical manifestations. Intramedullary tuberculomas are a rare form of central nervous system tuberculosis, accounting for only 0.2%-0.5% of all CNS tuberculomas. We report the case of a 28-year-old man with a history of non-Hodgkin lymphoma treated in 2014 who developed areflexic paraparesis with pyramidal signs and fecal incontinence shortly after initiating RIPE therapy for disseminated extrapulmonary tuberculosis. An MRI showed a nodular, intramedullary lesion, T10-T11 measuring approximately 1.2 × 0.8 × 0.7 cm, with intermediate signal on T2 and isosignal on T1, with complete peripheral and annular gadolinium uptake. A narrative review was performed using the keywords “intramedullary,” “spinal cord,” and “tuberculoma” in October 2024 including 120 articles; 26 were selected for full reading, with 22 included. Based on the previous literature, 35 patients were included. The majority of cases were from low- and middle-income countries (*n* = 33). 62% of the patients were male (*n* = 22), 57% of the patients had involvement in the thoracic segment (*n* = 19); 24% in the cervical (*n* = 9); and 18% in the lumbar (*n* = 6). Regarding treatment, the duration of treatment with RIPE varied across different studies. Surgical treatment was performed in 54% of cases (*n* = 19), while 46% were managed conservatively (*n* = 16). Tuberculosis remains a significant cause of disability in low- and middle-income countries, as well as a major contributor to paraparesis. The treatment of these cases exhibits considerable heterogeneity, both in terms of the definition and the choice between conservative management and surgical intervention, as well as the duration of antituberculosis therapy.

## Introduction

Tuberculosis remains a major global health burden and continues to rank among the leading causes of death worldwide, particularly in low- and middle-income countries [1]. Although central nervous system (CNS) involvement is a well-established manifestation of the disease, it represents a relatively small proportion of cases and is associated with significant morbidity [2]. Within this spectrum, intramedullary spinal cord involvement is exceedingly rare.

Intramedullary tuberculomas account for only 1-2 cases per 100,000 patients with tuberculosis [3] and comprise approximately 0.2%-0.5% of all CNS tuberculomas [4]. Most of the available evidence derives from isolated case reports and small series, with fewer than 200 cases described in the literature to date, predominantly involving the thoracic spinal cord [[5], [6], [7]]. While CNS tuberculomas occur in up to 10% of tuberculosis cases, the occurrence of intramedullary lesions—particularly in isolation—remains exceptional [2,4].

Historically, intramedullary spinal tuberculosis was first described in the early 19th century, and it continues to represent a minute fraction of CNS tuberculosis cases. Despite advances in diagnostic methods and antimicrobial therapy, tuberculosis remains an important cause of nontraumatic myelopathy and paraplegia in endemic regions [2] However, intramedullary tuberculoma is an uncommon presentation and may pose a significant diagnostic challenge due to its nonspecific clinical and radiological features.

Magnetic resonance imaging (MRI) is the modality of choice for evaluation, allowing precise delineation of lesion location, extent, associated edema, and contrast enhancement patterns, which are essential for diagnosis and therapeutic planning [3,5].

## Methods

This narrative review was conducted to provide an overview of the current evidence regarding intramedullary tuberculomas. A literature search was performed in PubMed up to October 2024 using combinations of the keywords “intramedullary,” “spinal cord,” “abscess,” and “tuberculoma.” The available literature consisted predominantly of case reports and small case series. A total of 22 articles were included in this review. Studies were selected based on their relevance to the topic and their contribution to the understanding of the clinical presentation, diagnosis, management, and outcomes of intramedullary tuberculomas. Given the narrative nature of this review, a formal assessment of study quality or risk of bias was not performed.

Data were extracted from the included studies and entered into a spreadsheet for descriptive analysis. Extracted variables included patient demographics (age and sex), treatment approach (surgical vs. nonsurgical management), and details of the therapeutic interventions. Descriptive prevalence analyses were performed to summarize the demographic characteristics, treatment patterns, and clinical features reported across the included cases.

### Human ethics and consent to participate declarations

This study was approved by the National Research Ethics Committee of the National Health Council of Brazil through Plataforma Brazil (CAAE 89619625.70000.5479) and by the Ethics Committee of Santa Casa de São Paulo.

## Case presentation

A 28-year-old man with a history of Ann Arbor Stage IV non-Hodgkin lymphoma, treated with 6 cycles of BEACOPP chemotherapy in 2015, presented to the emergency department approximately with 8 weeks with chronic diarrhea, weight loss (10% of body weight in less than two months), orthopnea, anorexia, and evening fevers. Imaging studies revealed a pleural effusion associated with conglomerated abdominal lymph nodes and moderate ascites.

A diagnosis of disseminated tuberculosis was established following thoracentesis of the pleural fluid, cervical lymph node biopsy, and bronchoalveolar lavage, which confirmed the causative organism. Treatment with RIPE therapy (rifampin, isoniazid, pyrazinamide, and ethambutol) was subsequently initiated.

Two weeks later, the patient presented to the emergency department with progressive weakness of the lower extremities and fecal incontinence, which had been present since the initiation of treatment and had worsened over the preceding days.

Neurological examination revealed that the patient was alert and oriented to time and place. Speech and language functions were preserved. Pupils were equal and reactive to light, with intact visual fields and extraocular movements. Cranial nerve examination was otherwise unremarkable. Motor examination demonstrated paraparesis associated with a thoracic sensory level at T10, with impaired superficial and deep sensation. The patient exhibited signs consistent with spinal shock, including absent patellar and Achilles tendon reflexes. Bilateral Babinski signs were present. At presentation, the patient was classified as **ASIA Impairment Scale Grade C,** with preservation of sacral function.

**Fig. 1:**
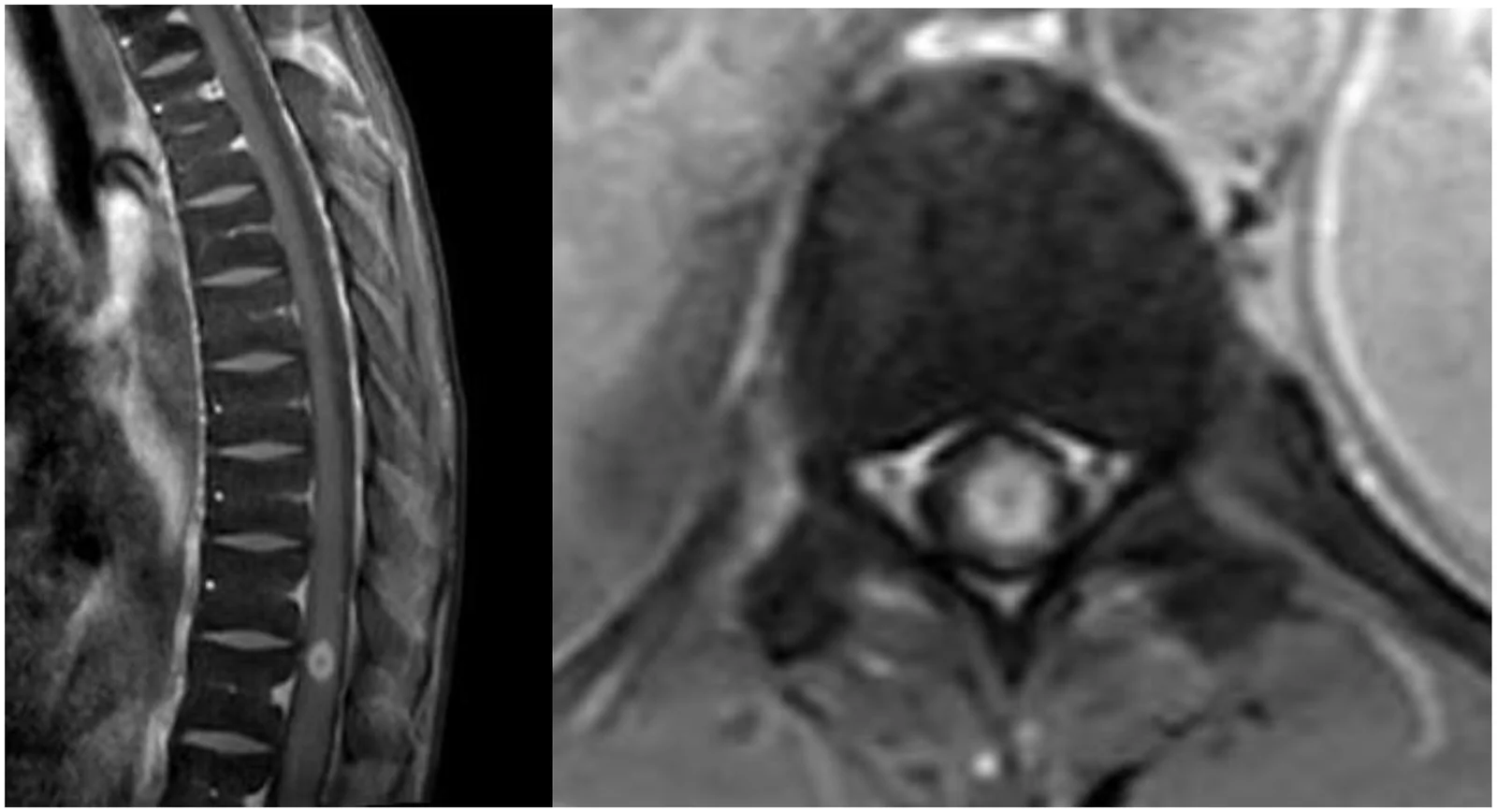
Sagittal and axial postcontrast T1-weighted MRI demonstrate a centrally located lesion with complete peripheral (ring-like) annular enhancement following administration of paramagnetic contrast, consistent with a rim-enhancing pattern. The enhancement delineates the lesion margins with a nonenhancing central component, suggesting central necrosis.

**Fig. 2:**
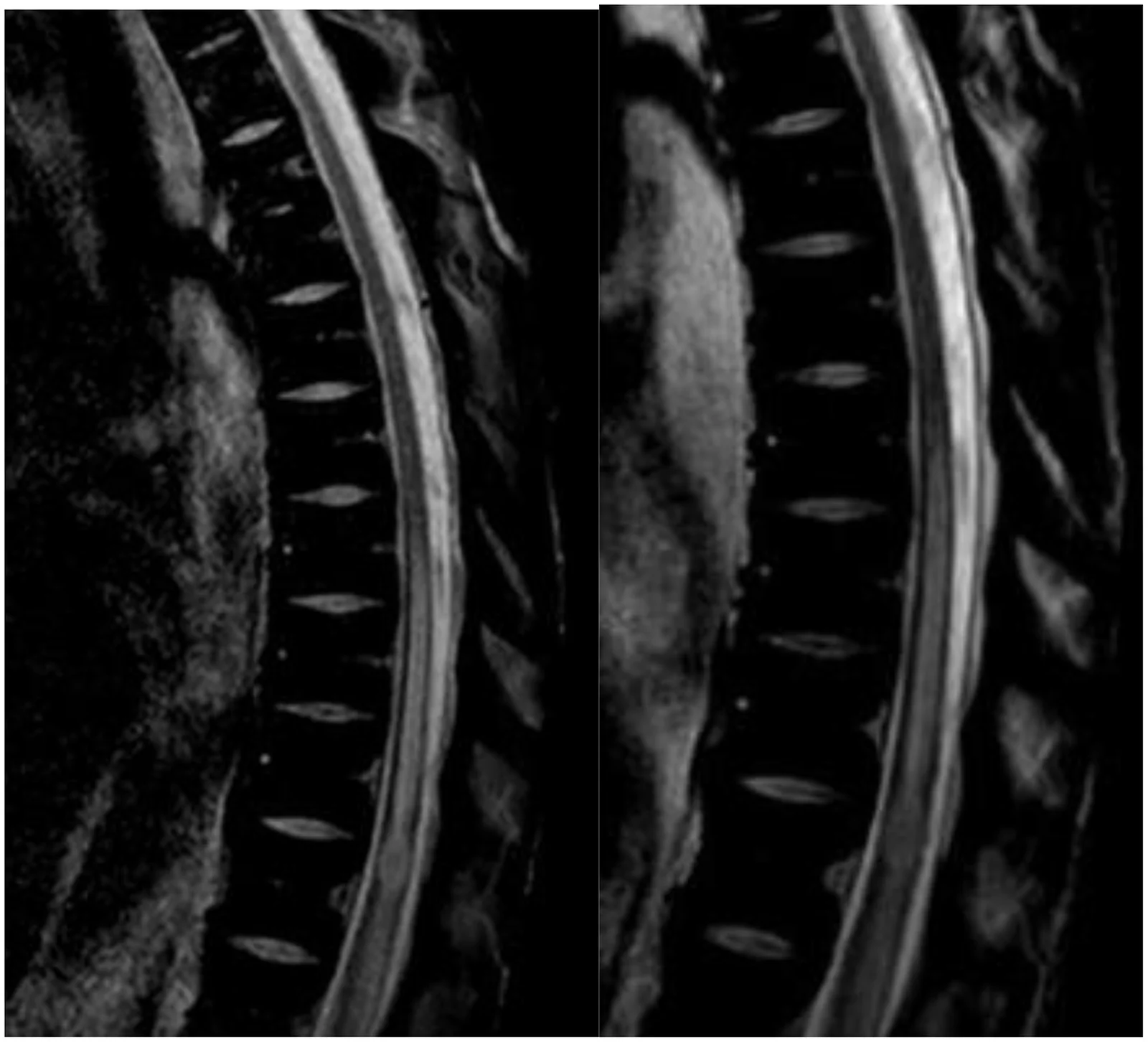
Sagittal T2-weighted MRI demonstrates a nodular intramedullary lesion at the T10-T11 level, exhibiting intermediate signal intensity. An associated extensive area of vasogenic edema is present, extending cranially from T9 to T12, resulting in significant cord involvement across multiple vertebral levels.

A MRI of the thoracic and lumbar spine was performed, revealing a well-defined, intramedullary nodular lesion at the T10-T11 transition (Fig. 1, Fig. 2), measuring approximately 1.2 × 0.8 × 0.7 cm. The lesion showed intermediate signal on T2 and isointense signal on T1, with complete peripheral and annular enhancement after gadolinium administration. Furthermore, the imaging also revealed an adjacent area of vasogenic edema extending from T9 to T12.

In the present case, the magnetic resonance imaging findings were considered consistent with a caseous intramedullary tuberculoma within the appropriate clinical context. The lesion was centrally located within the spinal cord at the T10-T11 level and exhibited a nodular morphology, adjacent spinal cord edema extending from T9 to T12, and complete peripheral ring enhancement following gadolinium administration, with a nonenhancing central component. The diagnosis was supported by the combination of disseminated tuberculosis, the presence of an intramedullary lesion with ring enhancement and a nonenhancing center, the absence of marked diffusion restriction, clinical improvement following antitubercular therapy combined with corticosteroids, and a significant radiological response on follow-up imaging.

The differential diagnosis included neoplastic lesions, particularly low-grade gliomas and ependymomas. Vascular etiologies were considered unlikely given the clinical presentation and imaging findings. Demyelinating disorders were also deemed less probable, and nutritional myelopathies were excluded based on the radiological appearance of the lesion. Given the patient's history of disseminated tuberculosis, an intramedullary tuberculoma was considered the leading diagnostic hypothesis.

Cerebrospinal fluid (CSF) analysis demonstrated hyperproteinorrhachia and hypoglycorrhachia without pleocytosis. In the presented clinical context, the spinal cord lesion was interpreted as a probable intramedullary tuberculoma.

## Our case treatment

It was decided to continue RIPE therapy, and dexamethasone 4 mg every 6 hours was initiated, with a follow-up imaging study scheduled to assess response to treatment.

Given the presence of a significant malabsorption syndrome for several days, the initiation date of tuberculosis therapy was reassessed. During the hospital course, concomitant HIV infection was ruled out; however, the patient was found to have a CD4 T-cell count of 156 and a CD4/CD8 ratio of 0.645. The hematology team was consulted and excluded tumor recurrence in the setting of disseminated tuberculosis, including pathological analysis of the cervical lymph node.

**Fig. 3:**
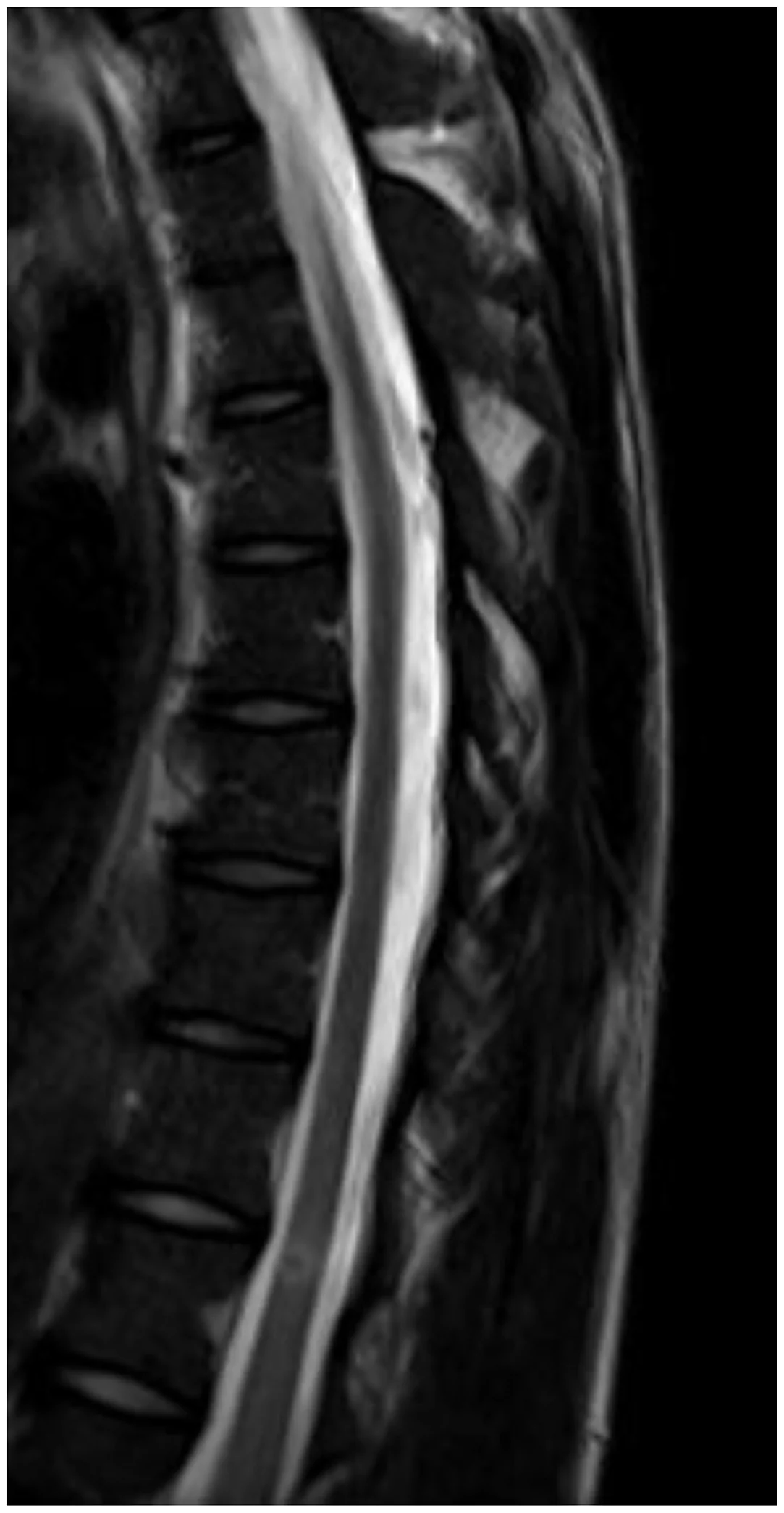
Sagittal T2-weighted MRI obtained after treatment demonstrates a marked reduction in the size of the previously described nodular intramedullary lesion at the T10-T11 level. The associated vasogenic edema has completely resolved, with normalization of the adjacent cord signal. These findings are consistent with a significant radiological response to the institute therapy.

The patient remained hospitalized for 23 days, with progressive neurological improvement, including recovery of lower limb strength, return of deep tendon reflexes, and resolution of fecal incontinence. He was discharged on RIPE therapy and a progressive corticosteroid taper, with continued clinical improvement. On outpatient follow-up after a month, he demonstrated lower limb strength graded 4+ and was undergoing physiotherapy, ambulating without assistance. Patient presented with **ASIA Impairment Scale Grade D in the first follow up.** Radiological improvement (Fig. 3) was consistent with the patient’s clinical recovery.

## Discussion

The MRI appearance of intramedullary tuberculomas changes throughout their evolution. In earlier stages, lesions are more often seen as solid nodules, typically hypointense on T1-weighted sequences and hyperintense on T2, with relatively uniform contrast uptake. As necrosis develops centrally, they tend to acquire a peripheral enhancement pattern after gadolinium administration, forming a well-circumscribed ring. On T2-weighted images, this configuration is usually represented by a central area of low signal intensity surrounded by a hyperintense rim. On T1-weighted sequences, the lesion commonly remains isointense relative to the spinal cord, with peripheral enhancement after contrast. Diffusion-weighted imaging (DWI) can be helpful in distinguishing these lesions from intramedullary abscesses, which more consistently demonstrate marked diffusion restriction. In our case DWI did not show marked diffusion restriction.

MRI is the imaging modality of choice for the diagnosis and follow-up of intramedullary tuberculoma. The most characteristic finding is a solid caseating tuberculoma demonstrating marked hypointensity on T2-weighted images (“T2 black sign”) with ring enhancement following gadolinium administration. In contrast, noncaseating tuberculomas typically appear hyperintense on T2-weighted images and exhibit homogeneous enhancement. On computed tomography, the “target sign”—consisting of central calcification surrounded by a hypodense area with peripheral ring enhancement—is suggestive, although not pathognomonic. Advanced imaging techniques may further improve diagnostic accuracy. Magnetic resonance spectroscopy often demonstrates a prominent lipid peak, reflecting caseous necrosis, which helps distinguish tuberculomas from primary neoplasms that more commonly exhibit elevated choline levels.

Imaging findings of tuberculomas vary according to the lesion's evolutionary stage. Noncaseous lesions tend to exhibit T2 hyperintensity and homogeneous enhancement. As caseation progresses, the central component may show a low or intermediate T2 signal—reflecting the compact caseous material—while enhancement becomes predominantly peripheral. In stages involving central necrosis or liquefaction, a more distinct nonenhancing center and ring enhancement may be observed. Thus, the pattern seen in this case—an intramedullary nodular lesion with complete peripheral enhancement and a nonenhancing central component—favors a diagnosis of caseous granulomatous lesion within the appropriate clinical context.

The radiological differential diagnosis for enhancing, expansile intramedullary lesions remains broad and includes primary spinal cord neoplasms, particularly ependymoma and astrocytoma, as well as intramedullary metastases, abscesses, demyelinating or tumefactive diseases, inflammatory myelitis, and vascular lesions. Extensive spinal cord edema that is disproportionate to the size of the enhancing lesion has been described more frequently in intramedullary spinal cord metastases [8] whereas marked diffusion restriction (high signal on DWI with low ADC values) is a characteristic feature of intramedullary abscesses [9]. Therefore, imaging findings should always be interpreted in conjunction with the clinical presentation, epidemiological context, and evidence of systemic tuberculosis.

Certain features in this case pointed toward an infectious-granulomatous etiology: the lesion’s small size relative to the extent of the edema, the ring-like enhancement with a nonenhancing center, the systemic context of disseminated tuberculosis, the absence of findings typical of primary spinal cord neoplasm, and the favorable clinical course following combined antitubercular and corticosteroid therapy. With appropriate antituberculous therapy, both clinical and radiological findings generally improve over time. Serial MRI typically demonstrates progressive reduction in lesion size and resolution of the surrounding edema, although residual contrast enhancement may persist because of ongoing inflammatory or reparative changes rather than active infection. Consequently, sequential imaging is essential for monitoring therapeutic response, documenting lesion evolution, and identifying potential complications while helping to avoid unnecessary surgical intervention in patients with a compatible clinical-radiological profile.

Follow-up MRI played a crucial role in supporting the diagnosis. The reduction of the intramedullary lesion and the resolution of adjacent spinal cord edema following specific treatment indicated a genuine therapeutic response, reinforcing the hypothesis of a tuberculoma and reducing the need for a diagnostic surgical procedure. In cases of intramedullary tuberculoma, serial radiological monitoring is particularly important, as the diagnosis often relies on integrating imaging findings, clinical context, systemic microbiological evidence, and the response to treatment.

Regarding the CSF findings, unlike tuberculous meningitis, intramedullary tuberculomas represent a localized granulomatous inflammatory response of the host. Therefore, marked inflammatory changes in the CSF are not necessarily expected. In this context, elevated CSF protein levels may occur due to disruption of the blood–spinal cord barrier, while the absence of pleocytosis does not exclude the diagnosis. In our case, the diagnosis was supported by the clinical presentation, characteristic imaging findings, histopathological confirmation of tuberculosis from lymph node biopsy, and subsequent clinical-radiological response to antituberculous therapy.

Our narrative review found 35 patient reports (Supplementary Table 1). Most of these cases (*n* = 33) originated from low- and middle-income countries. Most studies involved patients from India (*n* = 24), China (*n* = 5), the USA (*n* = 2), France (*n* = 1), and Taiwan (*n* = 1). The average age of the patients was 28 years, with a median of 28. Sixty-two percent of the patients were male (*n* = 22), while 37.14% were female (*n* = 13).

The most common location for these lesions reported was in the thoracic spinal cord, likely due to the vascular supply of this region and a higher incidence of hematogenous involvement. Other possible regions include the conus medullaris and cervical segments. Specifically, 57% of patients were involved in the thoracic segment (*n* = 19); 24% in the cervical segment (*n* = 9); and 18% were involved in the lumbar segment (*n* = 6).

In general, tuberculomas are accompanied by adjacent vasogenic edema, which can extend several levels above and below the main lesion. This was observed in the present case, where there was spinal cord involvement from T9 to T12 associated with a focal lesion at T10-T11. Complete peripheral and annular enhancement with gadolinium were noted on sagittal and axial T1-weighted images, along with extensive adjacent vasogenic edema, affecting the spinal cord between T9 and T12. This pattern is consistent with the caseous stage of tuberculoma, the most frequently identified in clinical practice, and correlates with the “target sign.”

Regarding treatment, in addition to the use of RIPE, the duration of treatment varied across different studies. Surgical treatment was performed in 54% of cases (*n* = 19), while 46% of cases were managed conservatively (*n* = 16). When reviewing the literature on the subject, it is generally indicated that surgery should be performed in cases refractory to medical treatment; however, upon reviewing the literature, it was found that most patients underwent surgery without prior clinical treatment [10].

Another interesting finding was the heterogeneity in the duration of RIPE treatment, as well as the inconsistent use of adjuvant corticosteroids. Most patients received RIPE for over 1 year, with some extending therapy up to 2 years. Conversely, only a minority of patients received adjuvant corticosteroid treatment.

As illustrated in this case, paradoxical worsening during antituberculosis therapy is a well-documented phenomenon, characterized by clinical or radiological deterioration after treatment initiation. This response is believed to reflect an exaggerated host immune reaction to mycobacterial antigen release following bacillary destruction, leading to intensified inflammatory activity. Consequently, previously existing lesions may enlarge, and new granulomatous formations can emerge, as observed in our patient [11,12]. This pattern has been consistently described in both intracranial and spinal forms of the disease. Importantly, such progression may be misinterpreted as treatment failure or drug resistance; however, in the absence of other supporting evidence, it generally does not require modification of the established therapeutic regimen.

## Conclusion

Tuberculosis remains a significant cause of disability in low- and middle-income countries, as well as a major contributor to paraparesis. However, the clinical presentation of intramedullary neurotuberculoma has been documented in only a few cases in the literature.

The treatment of these cases exhibits considerable heterogeneity, both in terms of the definition and the choice between conservative management and surgical intervention, as well as the duration of anti-tuberculosis therapy, which typically exceeds 1 year. The use of corticosteroids has also varied across studies. Despite this variability, the majority of patients have demonstrated clinical improvement. Therefore, further research is necessary to establish standardized protocols for the management of these patients.

## Author contributions

Swerts D. B., Marano C., Silveira G., Hazime N. contributed to planning the article, data collection, statistical analysis, conducting and writing the article. Riberio T., Penalva A. contributed to coordinating the article, as well as organizing the project.

## Patient consent

The authors confirm that written informed consent for publication of this case report and any accompanying images was obtained from the patient.

## Appendix Supplementary materials

Supplementary Table 1. Image, image 1 dummy alt text
