# Supplementary material for: Intramedullary neurotuberculoma in a patient with disseminated tuberculosis: A case report and a narrative review
Source: Radiol Case Rep. 2026 Aug 18;21(11):5440–5. doi: 10.1016/j.radcr.2026.07.076 (PMC13520988; doi:10.1016/j.radcr.2026.07.076)
Supplement: Supplementary file 1 [file mmc1.docx]

| DOI | N (Casos) | Localização Intramedular | Tratamento |
| --- | --- | --- | --- |
| 10.25259/SNI_1043_2021(11) | Female, India, 3 Y | T6 - T9 | T6‑T9 laminoplasty with intramedullary lesion decompression under neuromonitoring + Antituberculus treatment |
| 10.4103/2152-7806.153844(27) | Male, India, 25 Y | **T3** | Antituberculus treatment (ATT), started with isoniazid (H) 300 mg/day, rifampicin (R) 450 mg/day, pyrazinamide (Z) 1500 mg/day, and ethambutol (E) 800 mg/day daily for 2 months, followed by INH and RF for 10 months + Surgical Excision |
| [10.1007/BF02725669](https://doi.org/10.1007/bf02725669) (12) | Female, India, 6 Y | T4-T6 | ATT (isoniazid and rifampicin). Injection dexamethasone 0.5 mg/kg was administered intravenously as a single  daily dose for 10 days. This was followed with oral  prednisolone in the dose of 2 mg/kg/day, which was  later continued for a period of 6 weeks and tapered to  omission over the next 4 weeks. |
| 10.1038/sc.2009.58 (28) | Male, China, 42 Y | T6-T7 | Resection surgery + One year RHZE |
| 10.7759/cureus.28761(8) | Male, China, 55 Y | T7-T8 | RHZE along with supplemental pyridoxine. |
| 10.1055/s-0037-1599823(13) | Female, India, 49 Y | C3-C7 | Completed 7 months of ATT with RHZE. RH was continued for another 2 months |
| 10.1097/MD.0000000000008673(2) | Male, Taiwan, 33 Y | C3-C4 | RHZE for 6 months,  followed by RI for the next 12 months |
| 10.1016/j.radcr.2023.10.015 (14) | Male, USA, 30 Y | T2-T3 | Laminectomy from T1 to T3 and lesion resection was done |
| 10.1097/BLO.0b013e318065b73c (15) | Male, 27 Y  Female, 24 Y  Female, 20 Y | Block at D9 | Of the 3 patients with intramedullary involvement, one underwent myelotomy and decompression and died within 2 months of  surgery. The other 2 patients were treated nonoperatively with antitubercular therapy for a minimum of 1 year and showed complete neural recovery. |
| 10.14245/kjs.2015.12.1.5(16) | Female, 29 Y  Male, 1,5 Y  Male, 24 Y  Female, 35 Y  Male, 45 Y  Male, 28 Y  Male, 10 Y  Male, 42 Y  Female, 28 Y  Female, 37 Y  Male, 20 Y | T12-L1  T8-T10  T3-T4  T5-T6  T4-T5  T6-T9  L1 (conus)  C3-C6  T5-T8  L1 (conus)  T12-L1 (conus) | 3 patients were managed conservatively on ATT (Isoniazid (H), Rifampin (R), Etambutol (E), Pyrazinamide (Z), Streptomycin (S) and Moxifloxacin (M) for 3 months in intensive phase followed by two drug regime (HR) in continuance phase for 21 months. Oral Corticosteroid in tapering dose was given for the initial 6 weeks of intensive phase. Surgical excision of SIMT after decompressive laminectomy of the corresponding level was performed in 8 cases |
| 10.13107/jocr.2250-0685.271(17). | Male, India, 25 Y | T8 | Treatment with multiple medications (RHZE) and a short course of dexamethasone was initiated. After 12 weeks, the patient showed no signs of improvement. Surgery was performed to decompress the spinal cord with excision of the lesion, with good recovery after 6 months. |
| 10.1136/jnnp.2007.116012(18) | Male, India, 35 Y | T11–T12 | RHZE were given for the first 3 months followed by RH for  5 months. Corticosteroids were given for 6 weeks and gradually stopped. |
| 10.1097/00024720-200202000-00019. (19) | Female, Japan, 54 Y | C6 | Laminectomy and syringosubdural shunt placement were performed, and a tuberculoma was removed |
| **INTRAMEDULLARY TUBERCULOMA OF THORACIC SPINAL CORD IN AN APPARENTLY HEALTHY NIGERIAN: CASE REPORT** (6) | Male, Nigerian, 45 Y | T12-L1 | D12 and L1 laminectomy and posterior myelotomy with excision of the intramedullary  lesion. Treated with a 4-drug antituberculous regimen with physiotherapy and he made complete neurological recovery 8 months post-operatively. |
| 10.1080/10790268.2010.11689691 (20) | Male, China, 15 Y  Male, China, 40 Y | C4  T12 | Sem descrição |
| 10.1177/0883073808327837(21) | Female, India, 6 Y | T9-T10 | RHZE for 2 months, followed by RH for a total of 10 months. |
| 10.1016/j.ijtb.2015.07.012(22) | Male, India, 30 Y | C4-T8 | Anti-TB treatment, in addition, the patient underwent a C6-T8 decompressive laminectomy with median myelotomy and excisional biopsy of the lesion |
| 10.1038/sj.sc.3101896(23) | Male, China, 15 Y | C3-C4 | The lesion was excised totally. Postoperatively, the  boy was put on injection streptomycin 0.75 g daily for 1 month intramuscularly, isoniazid + rifampicin orally for another 12 months |
| 10.4103/1793-5482.143461 (5) | Male, India, 16 Y | L1 | He had complete recovery with conventional treatment of anti‑tubercular therapy and steroids, without any surgical intervention. |
| 10.1038/sj.sc.3100824(24) | Male, India, 15 Y | C5 | Rifampicin 450 mg, isoniazide 300 mg, pyrazinamide 1500 mg and ethambutol 800 mg with methyl prednisolone (MPS) 500 mg iv for 5 days followed by oral tapering dose of prednisolone over 2 months. |
| 10.17161/kjm.vol13.13674(25) | Male, USA, 71 Y | T7-T12. | RHZE therapy, supple­mented with adjuvant pyridoxine 50 mg/day for two months followed by 10 months of continu­ation therapy with rifampin and isoniazid alone |
| 10.1016/j.lpm.2010.10.030 (26) | Female, France, 27 Y | T2 | Corticosteroid therapy was initiated with a bolus of 15 mg/kg per day for two days. Neurosurgery with decompressive intent by laminectomy of T12-L1-L2 was decided upon. |
